# Complete Genome Sequence of Bacteriophage St11Ph5, Which Infects Uropathogenic *Escherichia coli* Strain up11

**DOI:** 10.1128/genomeA.01371-17

**Published:** 2018-01-11

**Authors:** Alla K. Golomidova, Eugene E. Kulikov, Vladislav V. Babenko, Elena S. Kostryukova, Andrey V. Letarov

**Affiliations:** a Research Center of Biotechnology of the Russian Academy of Sciences, Winogradsky Institute of Microbiology, Moscow, Russian Federation; b Federal Medical Biological Agency, Federal Research, Moscow, Russian Federation and Clinical Center of Physical-Chemical Medicine, Moscow, Russian Federation; c Moscow Institute of Physics and Technology, Dolgoprudnyi, Russian Federation; d Faculty of Biology, Lomonosov Moscow State University, Moscow, Russian Federation

## Abstract

Bacteriophage St11Ph5 was isolated from a sewage sample on a particularly phage-resistant uropathogenic *Escherichia coli* (UPEC) up11 host strain. It appeared to be closely related to bacteriophage G7C, isolated from horse feces; however, it carries a highly divergent host recognition module.

## GENOME ANNOUNCEMENT

The rapidly spreading antibiotic resistance of pathogenic bacteria is now globally recognized as one of the major threats to human health (1). Drug-resistant infections caused by *Escherichia coli* are one of the major concerns in urology (2). Bacteriophages are currently considered potential therapeutic agents that may comprise an alternative for antibacterial chemotherapy (3).

The uropathogenic *E. coli* (UPEC) up11 strain was isolated from a patient in the diagnostic unit of the Institute of Epidemiology in Moscow, Russia, in 2014. This strain has been identified as O5ab O-antigen producer (our unpublished data). The search in various environmental sources for bacteriophages against the up11 strain revealed that the rate of phage isolation for this culture was much lower than that for other *E. coli* strains, both uropathogenic and nonpathogenic, used as controls. Nevertheless, after substantial effort, a phage, St11Ph5, was isolated from a sewage sample. Electron microscopy evaluation indicated that the virion morphology of this phage was almost identical to that of phage G7C, previously isolated from horse feces (4), the cell recognition strategy of which has been studied in considerable detail (4, 5). Since phage G7C proved to be a useful model for investigation of the O antigen’s protective function (6), the putative relatedness of the phages St11Ph5 and G7C motivated us to determine the whole-genome sequence of St11Ph5 and analyze the proteins of the St11Ph5 adsorption apparatus. We believe that this information will eventually help us to unravel the mechanisms of the remarkable phage resistance of the *E. coli* up11 strain. Bacteriophage St11Ph5 stock was prepared, and DNA was extracted and sequenced using an Ion Proton sequencer system (Applied Biosystems, USA) with 400-fold coverage and a median read length of 182 bp. The raw reads from the run were then combined and filtered using the spectral alignment error correction tool SAET 3 (7). This yielded 157,282 reads with an overall 350-fold coverage. Primary assembly was conducted with Newbler version 3.0, resulting in a single contig representing the 72,439-bp genome sequence with 42.64% GC pair content. The obtained genome sequence was annotated using PROKKA 1.7 and manually edited.

The genome of St11Ph5 was highly related to that of phage G7C, with a nucleotide identity level of 92% over about 78% of the genomes, aligned by the blastN algorithm (http://www.ncbi.nlm.nih.gov). This allowed us to identify phage St11Ph5 as a member of the genus *G7cvirus*. This genus belongs to a wider group of N4-related phages that, from a phylogenetic point of view, apparently corresponds to the subfamily rank within the family *Podoviridae* in the order *Caudovirales*. However, the group of N4-related phages currently has no official taxonomic status adopted by the International Committee on Virus Taxonomy (ICTV).

The phage St11Ph5 genome codes for 90 open reading frames (ORFs). The analysis of the St11Ph5 genome annotation revealed that its genome organization is perfectly collinear with that of G7C. Therefore, we set the left end of the St11Ph5 chromosome at the same position as in G7C, since it has been confirmed experimentally (4). The adsorption proteins encoded by genes 76 and 77, corresponding to gp63.1 and gp66 in G7C, respectively, were identified. Although these proteins carry receptor recognition units that are completely different from those of G7C phage proteins, the overall architecture of the cell recognition devices appears to be well conserved in these viruses. This makes phage St11Ph5 potentially useful as a tool for the analysis of the strategies used by phages to penetrate the highly effective shield of *E. coli* up11 surface structures.

### Accession number(s).

The draft genome sequence of bacteriophage St11Ph5 has been deposited in DDBJ/ENA/GenBank under the accession number MG208881.
